# Legal approaches to risk of harm in genetic counseling: perspectives from Quebec and Qatar

**DOI:** 10.3389/fgene.2023.1190421

**Published:** 2023-07-28

**Authors:** Dimitri Patrinos, Mohammed Ghaly, Mashael Al-Shafai, Ma’n H. Zawati

**Affiliations:** ^1^ Centre of Genomics and Policy, School of Biomedical Sciences, Faculty of Medicine and Health Sciences, McGill University, Montreal, QC, Canada; ^2^ Research Center for Islamic Legislation and Ethics, College of Islamic Studies, Hamad Bin Khalifa University, Doha, Qatar; ^3^ Department of Biomedical Sciences, College of Health Sciences, Qatar University, Doha, Qatar

**Keywords:** genetic counseling, genetic counselors, risk of harm, policy, professional regulation

## Abstract

Genetic counseling is a fast-growing profession worldwide, with genetic counselors taking on increasingly comprehensive and autonomous roles in the healthcare sector. However, the absence of appropriate legal frameworks could potentially create risks of harm to the public. Legal recognition serves to protect the public from risk of harm by regulating the safe and competent practice of healthcare professionals. Genetic counseling is not legally recognized in most world jurisdictions. Examination of the legal status of genetic counseling in different jurisdictions and whether existing legal mechanisms are adequate to address potential risks of harm is therefore timely. This paper examines the different roles of genetic counselors in the Canadian province of Quebec and the state of Qatar, the authors’ respective jurisdictions. It considers the types of harms that may be created where appropriate legal mechanisms are lacking, considering the socio-political and legal differences between the two jurisdictions. Moreover, it examines the legal status of genetic counseling in Quebec and Qatar to determine whether these statuses appropriately address the identified risks of harm. The authors argue that existing legal frameworks are inadequate to address these risks and recommend that additional regulatory mechanisms be implemented to properly protect the public from risks of harm.

## 1 Introduction

Genetic counselors are healthcare practitioners with specialized education and training in medical genetics who counsel patients and their families on the medical, psychological and familial implications of genetic diseases and genetic testing options (Abacan et al., 2019; Biesecker, 2020). While there remains a shortage of genetic counselors in most countries compared to other healthcare professions (Stoll et al., 2018), the genetic counseling profession has grown significantly over the last few decades (Ormond et al., 2018; Abacan et al., 2019).

Indeed, genetic counselors are taking on increasingly comprehensive and autonomous roles in the healthcare sector. For instance, a growing number of genetic counselors are working in non-genetic clinical settings with non-geneticist physicians (see, e.g., Somers et al., 2014; Swanson et al., 2014; Shugar et al., 2017). This professional expansion, however, could potentially create risks of harm for the public if there are no appropriate regulatory mechanisms in place. Legal recognition serves to protect the public from risk of harm by regulating the safe and competent practice of healthcare professions. Currently, genetic counseling is not legally recognized in most world jurisdictions, though many are actively pursuing legal recognition (Ormond et al., 2018; Abacan et al., 2019). In jurisdictions in which genetic counseling is not a legally recognized profession, public protection mechanisms may be more limited, potentially exposing the public to risks of harm. Examination of the legal status of genetic counseling in different jurisdictions and whether their legal mechanisms adequately address these risks of harm is therefore timely.

In this paper, we will begin by describing the current roles of genetic counselors in the Canadian province of Quebec and in the state of Qatar, which represent the authors’ respective jurisdictions. We will then examine how, in the absence of appropriate regulatory mechanisms, these roles may create the potential for risks of patient harm. These risks may differ between the two jurisdictions due to their different socio-political and legal structures. Next, will examine the legal status of the profession in Quebec and Qatar respectively, to determine whether these legal statuses adequately respond to the risks of harm identified in these jurisdictions. Similarities and differences between Quebec and Qatar will be highlighted, both in terms of the legal status of genetic counseling and the manner in which their regulatory mechanisms address potential risks of harm.

## 2 Roles of genetic counselors and risks of harm in Quebec

Genetic counselors’ roles can vary across jurisdictions and even between institutions and departments (see, e.g., Pestoff et al., 2016; Dwarte et al., 2019; Marcus, 2019). In Quebec, genetic counselors work primarily in medical genetics settings (Fox and Secord, 2016). As in the rest of Canada, genetic counselors perform multiple roles. These include gathering and analyzing patients’ family and medical histories, conducting risk assessments, providing patients with information on genetic testing, explaining testing results, and providing psychological counseling and support (Lambert et al., 2022). While the majority of genetic counselors in Quebec work directly with medical geneticists, genetic counselors are increasingly working outside of medical genetics settings, such as oncology, cardiology and neurology. Moreover, they are also working outside of clinical settings, in areas such as research, education, and private industry (Fox and Secord, 2016; Hoskovec et al., 2018; Uhlmann, Hoskovec and Freivogel, 2020). In these settings, they are often the only practitioners with training and expertise in genetics (Shugar et al., 2017; Lambert et al., 2022), as non-genetics healthcare professionals often have limited knowledge of and training in genetics (Haspel et al., 2021). Even when working under the supervision of a medical geneticist, genetic counselors are becoming increasingly autonomous in their practice (Fox and Secord, 2016; Shugar et al., 2017; Lambert et al., 2022). For instance, in a survey on the clinical practices of genetic counselors in the Canadian province of Ontario, Shugar et al. (2017) found that respondents often worked with “high levels of independence with respect to clinical judgment and decision-making” (p. 100).

This increasing autonomy may create the potential for risks of public harm, given that genetic counseling is not a legally recognized profession in Quebec (Fox and Secord, 2016). Without legal recognition, anybody may practice genetic counseling or use the title of genetic counselor without having the proper skills and qualifications (Zawati, 2012; Zawati, 2018). Genetic counselors are increasingly performing complex tasks, such as interpreting complex genomic information and variants of uncertain significance (Shugar et al., 2017; Lambert et al., 2022). Furthermore, by virtue of their relationship with their patients, genetic counselors handle confidential and often sensitive patient information (Fox and Secord, 2016). The potential for patient harm if genetic counseling is not properly regulated is significant and may include inappropriate testing, cases of wrongful birth, wrongful sterilizations or pregnancies, inappropriate medical interventions, and financial and psychological harms (Fox and Secord, 2016; National Society of Genetic Counselors, 2016; Shugar et al., 2017). For instance, ordering inappropriate genetic testing may result in significant costs for patients (Brierley et al., 2012). Negligently performed prenatal genetic counseling, such as failure to provide accurate information about potential reproductive risks, may result in wrongful birth claims. These can result where the child is born with a disability and the child’s parents claim that the negligent counseling deprived them of the opportunity to terminate the pregnancy (O'Brien and Dugoff, 2018). In the United States, cases of harm involving genetic counselors have resulted in legal actions (National Society of Genetic Counselors, 2016).

## 3 Roles of genetic counselors and risks of harm in Qatar

In Qatar, genetic counselors perform similar roles to their counterparts in Quebec, including performing risk assessment of personal and family histories, counseling patients about genetic risks, interpreting test results and explaining them to patients (Ministry of Public Health, State of Qatar, 2022). Unlike in Quebec, however, genetic counselors may order genetic testing, an act restricted by law to physicians in Quebec (Medical Act, 2023, art. 31).

Genetic counselors play a key role within the mandatory Qatari premarital screening program, which was implemented in 2019 to decrease the incidence of certain genetic disorders, including cystic fibrosis, homocystinuria, thalassemia, sickle cell disease (and optionally spinal muscular atrophy) (Al-Shafai et al., 2022). The rate of consanguinity in Qatar has been estimated to be more than 50%, increasing the risk of having children with serious health or genetic conditions (National Development Strategy, 2011; Supreme Council of Health, 2011). Under this program, each party to the marital contract is required to submit to the marriage attestator or notary a medical certificate showing that the test was performed. It is important to note that only the test itself is mandatory. Regardless of the results, the to-be-married couple has the freedom to decide whether or not to proceed with the marriage. Indeed, while all to-be-married couples are required to be tested, only those at risk of having affected children will be referred for non-directive genetic counseling so that they are able to make an informed decision about their marriage plans. Potential preventative approaches, such as pre-implantation genetic diagnosis (PGD), are also discussed with couples, as they may wish to consider such options if they decide to proceed with their marriage plans.

Genetic counseling is usually introduced as a non-directive service, with the goal of providing neutral information, predictable outcomes and statistical probabilities without attempting to influence the decisions of the concerned parties. However, the implementation of the mandatory premarital screening program and the corresponding need for genetic counseling raises several potential risks of harm to the public. The scope of these risks can be quite broad, encompassing religious, ethical and socio-cultural considerations. Risks associated with the disclosure of confidential information and potential violations of privacy have been particularly emphasized by the literature (Budūr, 2008; Budūr, 2009; Naṣṣār, 2008). Such concerns were also expressed in public deliberations concerning the implementation of the Qatari premarital screening program, which examined its potential social impacts (Budūr, 2008; Budūr, 2009; Naṣṣār, 2008).

Indeed, one of the unique aspects of the genetic counseling process for discussing premarital screening results is the involvement of two parties, the prospective spouses. Both parties’ genetic information must be examined to determine whether it will be beneficial or harmful, from a genetic perspective, to proceed with the planned marriage (i.e., whether they are carriers for the diseases included in the test). Revealing information about one’s own genetic profile could also entail information about one’s genetically related relatives. Keeping in mind the social significance of the family institution in a Muslim-majority country like Qatar (National Development Strategy, 2011), disclosing the genetic information of one individual can have serious impacts on other individuals who belong to the same family or tribe. This may lead to potential social stigmatization for individuals and their families when their marriage plans are terminated because of unfavorable premarital screening results (Idrīs, 2017). The seriousness and magnitude of such harms are significant given that marriage, within the Qatari cultural and legal setting, is the only socially and legally sanctioned way to have children.

It is therefore clear that in both Quebec and Qatar, genetic counseling may pose risks of harm to the health and safety of the public if not properly regulated. These harms may include physical, psychological, and social harms. In Quebec, physical and psychological harms have been particularly emphasized, whereas social harms have been identified as significant in Qatar, given its socio-cultural and religious context. In light of these risks, we will now examine the legal status of genetic counseling in Quebec and Qatar to determine whether existing regulatory frameworks adequately address these risks and protect the public from risk of harm.

## 4 The legal status of genetic counseling in Quebec

Canada is a federation in which legislative powers are shared between the federal and provincial governments, the latter of which is responsible for the regulation of professions (Patrinos et al., 2020). Quebec, Canada’s second most populous province, uses a civil law system in matters of provincial authority, and legislation constitutes the primary source of law. In Canada, professions that are considered to pose a risk of harm to the health and safety of the public may become legally recognized through the adoption of legislation (Shugar et al., 2017; Patrinos et al., 2020).

In Quebec, the Professional Code governs the provincial professional system, which comprises those professions which have been legally recognized by the provincial government through the implementation of specific public protection measures. These include regulating access to specific professions, protecting professional titles, defining professional scopes of practice, and implementing public accountability measures (Patrinos et al., 2020; Lambert et al., 2022). Genetic counseling is not legally recognized in Quebec, however. This is true for all Canadian provinces, except for Manitoba, which has legally recognized genetic counseling through a limited form of delegation from physicians (Patrinos et al., 2020). The absence of legal recognition limits the measures that may be implemented to protect the public from risk of harm.

Three models of legal recognition are available under the Professional Code: 1) constitution of a professional order, 2) inclusion in an existing professional order, and 3) delegation (Patrinos et al., 2020). Under the first two models, control of access to the genetic counseling profession would be limited to individuals who meet the required criteria, training, and educational requirements. The professional title of genetic counselor would be protected by law, precluding non-genetic counselors from using this title or holding themselves out as genetic counselors. Moreover, under these two models, genetic counselors would practice with a legally defined scope of practice, delineating their activities and areas of professional practice. This could potentially include restricted activities, which are acts which may pose a risk to the public if not performed by a qualified professional. Finally, genetic counselors would be subject to continuous oversight and public accountability measures, such as public complaints procedures and disciplinary measures in the event of patient harm.

Delegation provides a more limited scope of public protection, specifically regulating the performance of specific acts, such as the communication of a diagnosis or the prescription of clinical tests or medication. It does, however, ensure some degree of public protection through the oversight of the performance of these acts (Lambert et al., 2022). In Manitoba, where delegation has been implemented, a physician may, under certain conditions, delegate the act of communicating a diagnosis relating to a genetic disease or disorder to a genetic counselor (College of Physicians and Surgeons of Manitoba General Regulation, 2018, s. 5.15(5)). While delegation ensures oversight of the performance of the delegated act, as the physician is responsible for its competent performance, delegation does not confer the same level of protection as other models of legal recognition, such as title protection and scope of practice (Patrinos et al., 2020).

None of these models apply to genetic counselors in Quebec, which limits legal recourse for patients in the event of harm. Under Quebec law, genetic counselors may, however, be considered service providers (Civil Code of Quebec, 2023, art. 2098; Zawati, 2018). Consequently, under Quebec’s civil liability rules, genetic counselors may be liable for any patient injuries caused by their acts or omissions (Zawati, 2018). While this offers some form of legal recourse in the event of patient harm, it is a reactionary measure. Legal recognition models represent more proactive approaches, through the implementation of measures to mitigate the potential for risks of harm, such as title protection, control of access to the profession, and mandatory continuing education and professional development programs. Indeed, these measures are implemented with the specific objective of mitigating risks of harm by ensuring that healthcare professionals provide their services in a safe, competent, and ethical manner, rather than by responding to specific instances of harm. These types of measures have been implemented in other jurisdictions where genetic counseling has been legally recognized, such as the United States, France, and South Africa (Patrinos et al., 2020). In the United States, 32 states (as of May 2023) have enacted licensure statutes, whereby genetic counselors must hold a governmental license to practice genetic counseling within their state (National Society of Genetic Counselors, 2023). Licensure serves to ensure that individuals have the relevant competencies to practice genetic counseling, thereby protecting the public from risks of harm that may occur where unqualified individuals provide genetic counseling services (Ormond et al., 2018; Abacan et al., 2019).

## 5 The legal status of genetic counseling in Qatar

Qatar is a unitary state bound not only by legal norms, but also by religious edicts rooted in the Islamic tradition. Indeed, Sharia, which refers to the body of Islamic religious law, is a principal source of legislation. ^1^Article 1 of the Qatari Civil Code (law no. 22 of 2004) states that judges shall rule as per the available statutory provisions (Almeezan, 2023). When such provisions are missing, however, the judge shall rule first in accordance with Sharia, then according to customary practice, and then according to the rules of justice. Where relevant statutory laws are missing, the management of possible harms ensuing from the mandatory premarital screening program and from the genetic counseling process should be made in alignment with Sharia.

The Ministry of Health recently implemented national registration requirements for genetic counselors in Qatar, thereby legally recognizing the genetic counseling profession. Under this framework, genetic counselors must apply for licensure to the Department of Healthcare Professions (DHP) (Ministry of Public Health, State of Qatar, 2022). The ministerial guidelines, like Quebec’s legislative scheme, prescribe educational requirements for the profession, provide a scope of practice statement for genetic counselors, and outline requirements for both competency validation and license renewal (Ministry of Public Health, State of Qatar, 2022). The importance of maintaining patient confidentiality is strongly emphasized in the guidelines, which state that genetic counselors must maintain confidentiality and make “every reasonable effort to ensure the security of written, verbal, and electronic patient information” (Ministry of Public Health, State of Qatar, 2022, s.1.2.4). Confidentiality is therefore an important value that underpins the genetic counseling process in Qatar.

The guidelines are silent, however, on how to deal with the risks of harm that may result from the genetic counseling process, including potential breaches of confidentiality. They state that genetic counselors must always “practice in accordance with relevant legislative, regulatory and policy guidelines”, but do not address specific public protection measures or legal recourses in the event of patient harm (Ministry of Public Health, State of Qatar, 2022, s. 1.3). As previously mentioned, in the absence of specific statutory provisions, Sharia, customary practices, and rules of justice are applied. Using the example of the harms associated with the disclosure of confidential information, we will describe how these harms may be addressed in a manner compatible with both Islamic values and Qatari legislation. Indeed, under religious principles, the agreed-upon and *prima facie* principle is that disclosing confidential information is prohibited and disclosing such information without a compelling reason may lead to legal penalties, unless the disclosure is permitted on exceptional grounds.

In the case of the above-mentioned mandatory premarital screening program, it seems particularly challenging to provide genetic counseling where the potential risk of harm can be mitigated or minimized. For practicing Muslim couples, the marital contract entails religious commitments with mutual rights and obligations. The legal enforcement of genetic testing raises concerns about the religiously protected right of autonomous individuals to decide what to do with their body and to choose their marriage partner. To mitigate this harm, genetic counselors in Qatar should make it clear to to-be-married couples that the mandatory nature of the testing is not meant to influence the religious validity of the marital contract. Furthermore, priority should be given to increasing public awareness of the significance of these genetic tests through public communications such as newsletters. Any laws and related executive orders associated with genetic testing should also be reviewed on a regular basis to determine if they are effective, if certain modifications are needed, or if they are still required. To avoid harms resulting from inadequate knowledge of the various Islamic perspectives on these questions, special training programs should be developed for genetic counselors to improve their religio-cultural competence. To meet Qatar’s need for genetic counselors and to have competent and religio-culturally sensitive counselors, Qatar University, the main national university in Qatar, has established a 2-year Master Program in Genetic Counselling in 2018 (Al-Dewik et al., 2018; Qatar University, 2023a). Graduates from the program, which includes courses, research, seminars, and clinical placements, are entitled to work as genetic counselors in Qatar (Qatar University, 2023a). Learning outcomes for the program include expertise in genetic testing, risk assessment skills, cultural competence, and ethical awareness and practice (Qatar University, 2023b).

## 6 Similarities and differences between Quebec and Qatar

The roles and responsibilities of genetic counselors in Quebec and Qatar are overall similar, despite the socio-cultural and legal differences between these two jurisdictions. This is consistent with research on the roles and responsibilities of genetic counselors globally, which finds similarities in the practice of genetic counseling across countries (Ormond et al., 2018). However, socio-cultural differences do play a role in how the genetic counseling process is shaped. In Qatar, this is illustrated by the role of genetic counseling within the national mandatory premarital screening program. Socio-cultural differences also shape the types of harms that may result from the genetic counseling process and how they may be viewed by society. Whereas physical and psychological concerns have been highlighted as potential risks of harm in Quebec, the social harms resulting from confidentiality breaches are considered more significant in Qatar.

These differences are not only limited to the roles of genetic counselors and the risks of patient harm in Quebec and Qatar, but also to the manners in which their respective legal systems respond to these risks. Whereas professions that pose a risk of harm to the public may become legally recognized in Quebec under its professional legislative scheme, this process has yet to be extended to genetic counselors. Consequently, there are few public protection mechanisms in place to mitigate potential risks of harm. Instead, patients who suffer injury from the genetic counseling process must use the legal recourses available under Quebec’s general civil liability rules. Though they afford some form of protection, these rules offer more limited protections and are more reactive than proactive.

Unlike Quebec, Qatar’s Ministry of Public Health has recently legally recognized the genetic counseling profession through the adoption of national guidelines. While these guidelines provide licensure requirements and create obligations for genetic counselors, including the obligation to maintain patient confidentiality, they do not provide information on how to deal with potential risks of harm.

## 7 Conclusion and recommendations

Genetic counseling is a rapidly growing and evolving profession. Genetic counselors are becoming increasingly autonomous and taking on more comprehensive roles in healthcare systems across the world. This professional expansion, without appropriate regulatory mechanisms to protect the public, may create risks of harm. This is true of the Canadian province of Quebec, where the lack of legal recognition limits the measures that can help protect the public from risk of harm. In Qatar, genetic counseling has recently been legally recognized by the Ministry of Public Health.

While the types of harm may differ between these two jurisdictions, the potential for risk of harm warrants the implementation of public protection measures. In Quebec, we recommend that genetic counseling be legally recognized and subject to the public protection mechanisms set out in the Professional Code. The potential risks of harm engendered by the unregulated practice of genetic counseling are sufficiently compelling to warrant legal recognition. The legal *status quo* in Quebec, whereby instances of patient harm are governed by ordinary civil liability rules, do not sufficiently protect the public from harm. Legal recognition is therefore required to ensure the safe, competent, and ethical provision of genetic counseling services in the province.

While genetic counseling is now a legally recognized profession in Qatar, the current regulatory framework does not provide for mechanisms to adequately address potential risks of harm. We recommended that the ministerial guidelines provide for mechanisms to ensure a wider ambit of protection for the public. We also recommend tailoring training programs to improve genetic counselors’ knowledge of the Islamic perspectives on risks of harms, thereby avoiding harms which could result from communicating incorrect or incomplete information during the genetic counseling process.

In summary, while the types of risks of harm may differ due to legal and socio-cultural differences, the case for the legal recognition of genetic counseling and for the implementation of public protection measures in both Quebec and Qatar is compelling. Whether they are implemented through legislation or through the application of religious and cultural norms, these measures are necessary to ensure safer and higher quality genetic counseling services for patients.

## Footnotes

1 See also the definition provided by Ghaly (2016): “An Arabic word which literally means way, road, or path to a source of water. In Islamic literature, this literal meaning was metaphorically employed to mean the way assigned by God for humanity to achieve success in this life and in the Hereafter, however with wide differences in the modern discussions about the exact scope of this term” (p. 423).
